# Litigation Friends or Foes? Representation of ‘P’ before the Court of Protection

**DOI:** 10.1093/medlaw/fww016

**Published:** 2016-12-22

**Authors:** Alexander Ruck Keene, Peter Bartlett, Neil Allen

**Affiliations:** 1 39 Essex Chambers, London, Honorary Research Lecturer at the University of Manchester, Manchester, UK, Wellcome Trust Research Fellow, Kings College London, London UK; 2 Faculty of Social Sciences, University of Nottingham, Nottingham, UK; 3 39 Essex Chambers, London, Senior Lecturer, School of Law, University of Manchester, Manchester, UK

**Keywords:** Best interests, Capacity, Court of Protection, Legal representation, Litigation friends

## Abstract

This article argues that, properly analysed, the common law and the European Convention on Human Rights (ECHR) march hand in hand with the provisions of the Mental Capacity Act 2005 (MCA 2005) so as to impose a set of requirements on litigation friends acting for the subject of applications of proceedings before the Court of Protection (‘P’) which are very different to those currently understood by practitioners and the judiciary. The authors examine critically current practice and procedures and provide a set of proposals for reforms.

## 1. Introduction

Ordinarily, a lawyer who submitted entirely the opposite of what she knew her client to wish would face, at best, professional sanctions, and at worst, a claim for negligence. In the Court of Protection, however, current practice would tend to suggest that there are circumstances where the lawyer must indeed argue against their client’s wishes. It is ironic and is (or should be) a cause for concern that they are doing so in relation to the most vulnerable of clients, and do so on the basis of instructions given by a person—a ‘litigation friend’—contending that they are acting in the best interests of the individual concerned.

This article examines why this situation has arisen, why it is unacceptable, and what steps can be taken to remedy it. Its central thesis is that, properly analysed, the common law and the European Convention on Human Rights (ECHR) march hand in hand with the provisions of the Mental Capacity Act 2005 (MCA 2005) so as to impose a set of requirements on litigation friends acting for the subject of applications of proceedings before the Court of Protection (‘P’^1^) which are very different to those currently understood by practitioners and the judiciary.

By way of context, the Court of Protection is established by the Mental Capacity Act 2005 to administer and adjudicate matters arising under that Act. Much of its work involves routine and uncontroversial applications relating to property and affairs (for instance, the appointment of deputies to administer the affairs of those who lack the capacity to do so), usually a purely administrative processes in which neither lawyers nor litigation friends are generally involved. Even for matters requiring a formal court decision, P is often not joined to all proceedings.^2^ This raises its own—important—questions,^3^ but they are not the focus of the present article. Our central concern is instead with cases where P is made a party to an application, and how those who are appointed to represent them discharge their duties. In principle, that can occur in any action before the court, but most frequently, these are cases involving personal welfare matters, including for example where P will live, what medical decisions are to be taken relating to P, or whether other individuals will be precluded from having contact with P—matters on which the views of P appear to be manifestly relevant. The discussion in this article is limited to these welfare cases. While the issues of relevance in this article also arise in cases involving P’s property and affairs, they are at their starkest, at least so far, in health and welfare matters.

Whenever P is a party, and if they do not have the capacity to conduct the proceedings, then at present a litigation friend must be appointed.^4^ Most frequently, this will be the Official Solicitor, but in principle it can be anyone appointed by the court, and sometimes others such as family members fulfil this role. The key question for this article is how that role is to be understood, vis-à-vis the present and past views of P. The Official Solicitor is almost invariably represented by counsel in the proceedings. In principle, other persons acting as litigation friends could represent P in person,^5^ although it seems this rarely if ever happens: in practice, litigation friends are represented in court by lawyers. In Section V, the article addresses the specific position of lawyers, both as instructed by litigation friends and, in due course, as so-called Accredited Legal Representatives (ALR) appointed to represent P directly without the interjection of a litigation friend.^6^

## II. WHY DOES REPRESENTATION MATTER?

‘Representation’ differs from either ‘participation’ or ‘presentation’ and that distinction illustrates the difference between what is, and what should be, the role of the litigation friend. Put at its simplest, it is the difference between steps being taken for P (either by the court or by a person appointed by the court) to put their views before the court, and steps being taken on P’s behalf actively to argue P’s case before the court. A litigation friend acts on P’s behalf;^7^ a task of an ALR will be to represent P.^8^ For the purposes of this article, and save where specifically identified, we proceed on the basis that both litigation friends (and the lawyers they instruct) and—in due course—ALRs are, or should, be representing P.

There are at least three overlapping jurisdictional reasons why representation (as opposed to mere participation or presentation) matters. The first is the domestic law, in particular the MCA 2005 itself, as bolstered by the common law demands of procedural fairness. The second is the requirements of Articles 5, 6, 8, and 14 ECHR. The third is the expectations of the United Nations Convention on the Rights of Persons with Disabilities (‘CRPD’).

### A. Domestic Law

The MCA 2005 does not impose express requirements as regards representation before the Court of Protection, leaving this to the Court of Protection Rules (COPR) (addressed further in Section IV(A) below). However, it is clear that the principles contained in section 1 MCA 2005 are equally applicable to the decision-making processes of the court itself, both as regards determination of whether P has the relevant decision-making capacity and, if they do not, what decision to take on their behalf in their best interests.

If P lacks capacity, it is also clear that decisions must be made in his or her ‘best interests’ as defined in section 4 MCA 2005, and that this section binds the Court as much as any other decision-maker. That section includes a requirement that P’s beliefs, values, past and present wishes and feelings must be considered. It also, in section 4(4) MCA 2005, requires that the court ‘must so far as reasonably practicable, permit and encourage the person to participate … as fully as possible in any … decision made for him’. Certainly, participation extends well beyond representation, and if the applicant (eg the local authority or health trust) has implemented the Act properly, P will have been involved throughout the pre-court discussions and procedures, and those views should have already been given serious consideration by the relevant experts in the case. No doubt these views, like much other background information, will be presented to the court, along with the comments by the learned experts as to the weight that should be accorded to those views.

It is in court, however, where the actual decision will be taken, and section 4(4) MCA 2005 thus requires that P be able to participate as fully as possible at this stage. Involvement in pre-court processes is not sufficient: that is not where the decision is being taken. Participation in the decision must mean more than being talked about in the forum where the decision is made; it must mean being able to be heard, and (crucially for present purposes) to have arguments presented in defence of one’s views.

The discretionary nature of much of the best interests test makes such direct involvement a particularly important element of the scheme of the MCA 2005. ‘All relevant circumstances’ are to be included (section 4(2) MCA 2005), with no guide as to how different factors are to be weighed against each other. P’s wishes and feelings are only one factor for consideration, and even here, considerable discretion exists as to the weight to be accorded to them, based on P’s capacity, the strength and consistency of P’s views, the impact on P of knowing that his or her wishes and feelings will be disregarded, the rationality of P’s wishes, and the consistency of those wishes with P’s overall best interests.^9^ There may be much, therefore, for P to want to make submissions about. The urgency of this is buttressed by the seriousness of many of the decisions, including serious medical treatments, restraint, detention in a care home or similar environment, being forced to move from one's own home, or being prevented from contact with family members. The court has, occasionally, noted the risk that misapplication of the Act can marginalize service users and their families, through overzealous interpretations by professional carers, both of best interests and incapacity.^10^ To protect against this, the professional evidence must be appropriately tested in court, and one way to provide for that is by allowing P appropriate scope to advance his or her arguments.

This is entirely consistent with the common law, whose cardinal principles regarding procedural fairness have re-emerged as the primary source of legal authority on the matter.^11^ The common law rationale and philosophical underpinnings were eloquently outlined by Lord Reed in *R (Osborn) v Parole Board*,^12^ emphasizing the importance of respect for the individual to be affected by the decision-making process. These underpinnings, it is contended, are equally pertinent to Court of Protection proceedings; but it is not just about the general principles of common law, but also the scheme of the MCA itself.

It would be difficult to argue that a ‘P’ with clear wishes and feelings as to what should happen to them does not have anything relevant to say to the decision(s) to be taken as regards them by the Court of Protection. In consequence, ‘[r]espect entails that such persons ought to be able to participate in the procedure by which the decision is made’.^13^ If a person is said to require a litigation friend in order to participate in the procedure before the Court of Protection, then, as amplified further below, procedural fairness dictates that the litigation friend be (and be seen by P to be) representing P, rather than discharging any other functions.

### B. The ECHR

Proceedings that determine an individual’s mental capacity engage Articles 6^14^ and 8^15^ of ECHR.^16^ The right in Article 6 is to *effective* access to a court. The State ‘must show particular vigilance and afford increased protection in view of the fact that such individuals’ capacity or willingness to pursue a complaint will often be impaired’.^17^ It is trite that, while procedural arrangements can be made so as to secure the good administration of justice and protect the health of the person concerned, such measures should not affect the very essence of the individual’s right to a fair trial guaranteed by Article 6(1).^18^

Strasbourg has recognized the need for there to be no conflict of interest between the subject of deprivation of capacity proceedings and any person appointed to act as their litigation friend.^19^ The ECtHR has also emphasized that in the case of ‘mentally disabled persons the States have an obligation to ensure that they are afforded independent representation, enabling them to have their Convention complaints examined before a court or other independent body’.^20^

In cases concerning detention on the basis of mental disorder, engaging the procedural rights afforded by Article 5(4), ‘when a mental patient is not fully capable of acting for herself on account of her mental disabilities, by definition the compensatory safeguards to which the State might have recourse in order to remove the legal or practical obstacles barring such a person from being able to benefit from the procedural guarantee afforded by Article 5 § 4 may well include empowering or even requiring some other person or authority to act on the patient’s behalf in that regard’.^21^

However, the ECtHR has so far—and disappointingly—failed to spell out the positive duties upon those acting as litigation friends to secure the rights of those they represent under Articles 5, 6, 8 (and, in conjunction with each of these, Article 14). The closest that the Court has come was in *RP v United Kingdom*,^22^ in which it held that it was not in the protected party (the mother)’s best interests for an unarguable case to be advanced on her behalf as to her daughter’s interests in care proceedings. This decision is entirely inconsistent with those outlined above, and singularly fails to grapple with the issues. More pertinently, it is clearly not applicable in proceedings before the Court of Protection when it comes to representing P—the subject of the proceedings whose interests are most vitally at stake.

### C. The CRPD

A third potential source of obligations that are relevant here is the CRPD; in particular Article 12 (equal recognition before the law) and Article 13 (access to justice). The CRPD is the current wild card in mental disability law. The United Nations Committee on the Rights of Persons with Disabilities has recently published a General Comment on Article 12, stating that any use of disability, directly or indirectly, as a defining feature of incapacity is in violation of the CRPD. The Committee expressly states that this includes functional tests of capacity^23^—the basis of the MCA 2005. Instead of capacity thresholds, differing abilities are to be addressed through the provision of supported decision-making. A similarly restrictive interpretation has been made precluding deprivation of liberty or enforcement of treatment when these purport to be justified in whole or in part by disability.^24^ There can be little doubt that if these interpretations are correct, fundamental change to English law, including the MCA 2005, will be required.

However, whatever the merits of the Committee’s approach, and its views are controversial,^25^ there is no consensus on how to implement it in the short term. This means that, for better or worse, the MCA 2005 may well be with us for some time to come. The present article acknowledges that reality, and is written on the assumption that whatever the formal requirements of international law, the present legal structure is unlikely to undergo fundamental change in the near future. At the same time, the values of the CRPD, including most notably the ethos of empowerment of persons with disabilities, and their right to full social and legal equality and participation, provide an important set of values underpinning this article. As such, it can perhaps be seen as part of a process of edging English law towards closer compliance with the CRPD, while acknowledging that full compliance, whatever that is taken eventually to mean, is still a long way off.

## III. EFFECTIVE REPRESENTATION: SECURED OR SCUPPERED?

None of the issues set out above would arise if a litigation friend—or, in due course, an ALR—were not inserted between P and their legal representative (or between P and the court if P were to act as a litigant in person). Different issues might arise in terms of the *ability* of P actually to give instructions or to act as a litigant in person, but their *legal* capacity to act as a party would not be impaired.

However, in proceedings before the Court of Protection, P, where joined, is routinely deprived of that legal capacity, on the basis that they lack the capacity to conduct the proceedings. Indeed, until July 2015, the default position was that P—if joined—required a litigation friend,^26^ unless P could, in essence, prove that they had capacity to conduct the proceedings.^27^ The position is now somewhat less egregious,^28^ but—in practice—it is very unusual for P to be considered to have capacity to conduct the proceedings. Indeed, judges have traditionally expressed a considerable degree of doubt as to whether a person who lacks the capacity to make a decision can ever have the capacity to conduct proceedings relating to that decision.^29^

There are signs that judges are beginning to be more sophisticated in this regard.^30^ However, it is striking just how different practice before the Court of Protection is to that before the Mental Health Tribunal, in which it is widely recognized that the threshold of capacity to give instructions to a legal representative is set low, at least when it comes to challenging detention.^31^ It is not merely a rhetorical question to ask as to whether there is any functional difference between such proceedings and at least some categories of welfare proceedings before the Court of Protection.

Further, linked to this, it is striking that there has been no apparent consideration to date of the impact of section 1(3) MCA in the context of the determination of capacity to conduct proceedings. If it is indeed the case that a litigation friend/ALR is standing in the shoes of P and making decisions as to the conduct of the proceedings on a best interests basis (an assumption that is questionable, but appears to be generally accepted—see further Section IV(B)(2)), then the logical corollary of that must be that, before the stage is reached of appointing that litigation friend/ALR on the basis P lacks capacity to make those decisions, all practicable steps must have been taken to support P to make those decisions without success. Otherwise section 1(3) MCA cannot have been complied with, and the litigation friend/ALR can have no place making best interests decisions on P’s behalf. This undoubtedly opens the door to some fruitful exploration of the steps that may be required to ensure that only those individuals who truly lack capacity to conduct the proceedings are given the ‘protection’ of a litigation friend or ALR. Some of the steps that may be required in this regard are examined at the conclusion of this article.

## IV. LITIGATION FRIEND OR FOE?

### A. Historical Background

It was recognized at the time that the new Court of Protection was established that two very different court models were being blended into one: the first, the inherent jurisdiction exercised by the High Court in health and welfare cases;^32^ and the second the much more informal jurisdiction exercised by the ‘old’ Court of Protection.^33^ The first was recognized as offering a ‘very high level of protection for the person who lacks capacity and his or her interests’, including that they would normally be joined as a party and provided with a litigation friend.^34^ The second was very much more informal, with an ‘inquisitorial’ flavour, without the naming of people as ‘defendants’:

They do not require the person who lacks capacity to be visited or interviewed by strangers whose involvement the person who lacks capacity may not welcome. They do not necessitate the involvement of lawyers in cases where there is no particular legal dispute.^35^

The (then) Department of Constitutional Affairs (DCA), when consulting upon the draft rules for the new Court of Protection, expressly sought to draw analogies with the range of ways in which children might be involved in family proceedings, and placed a significant emphasis upon court reports,^36^ as ‘a valuable alternative to full, formal representation by a ‘litigation friend’ and lawyer which is arguably more suitable to adversarial litigation but which has had to be used in the High Court to date’, allowing ‘a flexible scheme where the person who lacks capacity is offered all appropriate protection within the court case but is not forced to accept (or pay for) a level of involvement which is distressing, unwanted or unnecessary’.^37^ The thinking behind these proposals led ultimately to the position noted above that prevailed until July 2015—i.e. that, unless an order was specifically made, P would not be joined as a party.

The consultation on the draft rules did not identify when P should be joined as a party or pose any questions as regards the *duties* of a litigation friend when P was joined. There is surprisingly little in the COPR or in the accompanying Practice Directions that identifies the duties of litigation friends or (in due course) of ALRs. Both must fairly and competently conduct proceedings on behalf of P,^38^ but the COPR, the accompanying Practice Direction 17A and indeed the MCA do not provide further guidance as to how such litigation friends or (in due course) ALRs are to act on P’s behalf.

It is therefore likely that the practice of those appointed where P is a party will continue as it has done to date in the Court of Protection. To this end, it is important to understand how that practice has evolved; an evolution which, impressionistically, is more than a little haphazard,^39^ drawing in significant part, upon practice and procedure from very different contexts without recognizing that these contexts are frequently far removed from the Court of Protection.^40^

It is possible to identify three (if not) four essentially separate strands of practice and authority as regards the appointment of litigation friends^41^ which has fed into current practice in the Court of Protection.

- The first derives from civil litigation where the person concerned is either a claimant or a defendant.
- The second derives from litigation involving children where the child concerned is not (necessarily) a party but is affected—for instance, adoption, wardship, or care proceedings.
- The third strand of practice and authority relates to the role of the Official Solicitor.
- The fourth relates to legal representatives appointed by the Mental Health. Tribunal^42^ to act for patients who lack the capacity to conduct proceedings before that Tribunal.

The practice in those different contexts have not previously been the subject of any prior comparative examination, and it is therefore necessary to outline them in a little detail to make clear why—on a proper analysis—they provide so unsatisfactory a foundation for practice before the Court of Protection.

#### 1. Civil Litigation

That a person who is incapable (whether by reason of age or mental disability) of conducting proceedings must act by a person with authority to give instructions on their behalf is a very long-established principle.^43^ Prior to the end of the nineteenth century, well-developed practices and procedures had been established at common law or set down in rules of court.^44^ These principles were repeated in twentieth century authorities^45^ and the relevant rules of court, being neatly summarized thus by Sir Robert Megarry V-C in *Re E (Mental Health Patient)*:^46^

The main function of a next friend appears to be to carry on the litigation on behalf of the plaintiff and in his best interests. For this purpose the next friend must make all the decisions that the plaintiff would have made, had he been able. The next friend may, on behalf of the plaintiff, do anything which the Rules of the Supreme Court require or authorise the plaintiff to do, though the next friend must act by a solicitor: see R.S.C., Ord. 80, r. 2.^[47]^ It is the next friend who is responsible to the court for the propriety and the progress of the proceedings. The next friend does not, however, become a litigant himself; his functions are essentially vicarious.

It should be emphasized that the principles identified above were developed in the context of civil litigation and/or other litigation in which such a person sought to assert or defend their rights, rather than in the context of litigation relating to that individual. This meant that at least part of the rationale for imposing a requirement for a next friend/guardian ad litem was for the benefit of the other parties, not least given the difficulties in recovering costs against a person without capacity to conduct proceedings.^48^ In *Masterman-Lister v Brutton & Co, Masterman-Lister v Jewell and Another*,^49^ for instance, Kennedy LJ noted that ‘[i]n the context of litigation, rules as to capacity are designed to ensure that plaintiffs and defendants who would otherwise be at a disadvantage are properly protected, and in some cases that parties to litigation are not pestered by other parties who should be to some extent restrained’.^50^ To similar end, Chadwick LJ noted that a ‘defendant is entitled to expect that he will not be required to defend proceedings brought against him by a person of unsound mind acting without a next friend’.^51^ *Masterman-Lister* has been the subject of high judicial endorsement since the enactment of the CPR,^52^ so there is no reason to consider that these principles are not still applicable, at least in the context of civil litigation.

Part 21 of the CPR, which now governs the appointment and duties of litigation friends, does not elaborate upon their obligations. CPR r.21.2(1) makes clear that a protected party must have a litigation friend to conduct proceedings on their behalf. The editors of the *White Book* note^53^ that the meaning of this phrase ‘is not elaborated in the rules but doubtless would include doing anything which in the ordinary conduct of any proceedings is required or authorised by a provision of the CPR to be done by a party to the proceedings’. They continue that the duty of a litigation friend ‘is no longer expressly defined as it was in the former Practice Direction supplementing this Part. However, the duty must be to fairly and competently conduct proceedings: see r.21.4(3)(a)’.^54^ The Court of Appeal in *RP v Nottingham City Council and the Official Solicitor (Mental Capacity of Parent*) endorsed the advice given by (then) Peter Jackson QC to the Official Solicitor as to his obligations when acting on behalf of a protected party,^55^ in which (inter alia) Peter Jackson QC noted that ‘[t]he meaning of ‘conduct proceedings on behalf of’ is not further defined, but the statement encapsulates the two magnetic influences upon the conduct of the litigation friend. The prime motivating factor is beneficence — acting for the parent's benefit. The second is competence — acting according to proper professional standards’.

A key consequence of the nature of the obligations upon a litigation friend acting for a protected party identified above is that the courts have made clear in the context of ‘conventional’ proceedings that such a litigation friend is not bound by the wishes and feelings of the protected party in the same way that a legal representative is (as a general rule) bound to follow the instructions of their client. This approach was upheld by the European Court of Human Rights in *RP v United Kingdom*,^56^ a decision which, as noted in Section II(B) is both questionable as an interpretation of the ECHR and clearly distinguishable when it comes to proceedings before the Court of Protection.

#### 2. Proceedings Concerning Children

At least on occasion, the courts in the twentieth century appeared equally happy to apply the principles set out above to proceedings in which they were exercising a parental jurisdiction over children, rather than determining a conventional dispute between parties.^57^ Importantly, however, in such proceedings, there was an increasing recognition that the interests of the child and of justice required something else. Statutory provision was made, initially in adoption proceedings,^58^ and then in care proceedings,^59^ for the appointment of a guardian ad litem whose role was, in essence, to provide an independent second opinion for the court as to the proposals being advanced before it by those other than the child themselves.^60^

The statutory provisions for children’s guardians are now contained in section 41 Children Act 1989 and the FPR.^61^ As a general rule, a child cannot now be joined as a party to family proceedings in which they are a subject without a children’s guardian^62^ being appointed for them.^63^ The precise role that the guardian will play will depend upon whether the proceedings are ‘specified’ or not (broadly, ‘specified’ proceedings represent state interventions such as applications for care orders; ‘non-specified’ proceedings are private law proceedings involving the child’s parents).^64^ There are very detailed provisions set down in the FPR as regards the discharge by the children’s guardian of their duties including, perhaps most relevantly for our purposes, a specific obligation to make such investigation as may be necessary for him to carry out their duties.^65^ There also specific provisions about the advice that the guardian must give to the court including the wishes of the child in respect of any matter relevant to the proceedings including that child’s attendance at court; and the options available to it in respect of the child and the suitability of each such option including what order should be made in determining the application.^66^

Crucially, a guardian is not appointed to represent the child, but is rather appointed *for* the child.^67^ There may, further, be situations in which a child both has their own solicitor and a guardian. The former may well be bound to follow the child’s instructions,^68^ but the child’s guardian will nonetheless retain a role in placing before the court anything which they consider to be in the best interests of the child.^69^ This is not the place to engage in a detailed discussion of the merits of children’s guardians and/or the literature relating to securing the voice of the child within proceedings;^70^ for our purposes, the key points to note is that in proceedings concerning children, there is now a clear statutory recognition that:

- There are two roles in play: (1) a ‘conventional’ litigation friend to act *on behalf* of a child who is the subject of proceedings; and (2) a guardian to act *for* a child who is the subject of proceedings. As described by Munby J (as he then was), the guardian is ‘both the voice of the child and the eyes of the court’.^71^
- In order to properly protect and further the child’s best interests, children who are subject to proceedings^72^ require the benefit of *both* representation by a solicitor (instructed, where relevant, by a litigation friend) and a guardian.

#### 3. The Official Solicitor

The office of Official Solicitor to the Supreme Court dates back to 1875,^73^ although the office derives ultimately from the institution of the office of Solicitor to the Suitors’ Fund of the High Court of Chancery in 1828. The Official Solicitor performs duties ‘pursuant to statute, rules of court, direction of the Lord Chancellor, at common law, or in accordance with established practice’.^74^ Among those duties have included acting as guardian ad litem or next friend for both children and adults mentally incapable of acting in many types of proceedings, including civil litigation and family proceedings.^75^

Insofar as the Official Solicitor acted as (now) litigation friend for a party in civil proceedings incapable of acting on their own account, the principles that applied to him were, it seems, essentially the same as would apply to any other next friend/guardian ad litem: see *Re E*. The same would also go for the Official Solicitor acting for a protected party in family proceedings,^76^ as was recognized by the Court of Appeal in *RP v Nottingham City Council and the Official Solicitor (Mental Capacity of Parent)*,^77^ discussed above.

However, in relation to wardship cases involving children, the Official Solicitor seems traditionally to have had a rather wide role (or series of roles). As the Court of Appeal noted in *Re G (Minors) (Wardship: Costs)*:^78^

In the performance of his duties in wardship cases, the Official Solicitor is much more than a mere guardian ad litem. He is at once amicus curiae, independent solicitor acting for the children, investigator, adviser and sometimes supervisor. Perhaps the nearest analogy is that of counsel to a tribunal of inquiry, a relatively new office but a valuable one.

There is a remarkable dearth of equivalent authority in relation to the Official Solicitor’s role as guardian ad litem (and then litigation friend) in proceedings under the inherent jurisdiction to grant declaratory relief in relation to adults lacking the material decision-making capacity, the functional precursor to the welfare jurisdiction of the ‘new’ Court of Protection which came into effect in 2007. However, in one high-profile case, that of Anthony Bland, the Official Solicitor took it upon himself to ‘ensure […] that all relevant matters of fact and law are properly investigated and scrutinised before any irrevocable decision is taken affecting [the person], for whom he acts as guardian ad litem’.^79^ That role—going beyond representation into investigation and assessment—was implicitly endorsed by the House of Lords.

It is perhaps important to emphasize that the Official Solicitor has also traditionally been called upon to play a number of other roles in addition to (now) litigation friend of last resort, including those he discharges as the court’s own solicitor.^80^ In consequence, there may in some of the reported cases, have been degree of ‘mission creep’ in consequence, as hinted at in *Re G* above.^81^ In other words, the court will have called upon the Official Solicitor, when appointed as litigation friend, to carry out other functions, but without necessarily appointing him specifically to discharge those functions or recognizing that it has invited him to step outside the role of litigation friend.

There are a number of specific provisions relating to the Official Solicitor in the COPR; as regards the discharge of his duty as litigation friend; however, he is bound by the same rules (insofar as ascertainable) as any other litigation friend. We return to how the current Official Solicitor views his role below (Section IV(B)(2)).

#### 4. Mental Health Tribunals

It should, finally, be noted that there is (at least) one further setting in which a participant in the proceedings may be required to act by a person who they have not directly instructed. It is of relevance to the matters that we are considering because—as with proceedings before the Court of Protection—the person concerned is in a rather different position to a claimant or defendant in civil family proceedings. Rather, they are participants in a judicial procedure designed to vindicate their rights for ‘societal’ purposes.

Where a patient has not appointed a representative, the Mental Health Tribunal^82^ may appoint a legal representative where (inter alia) ‘the patient lacks the capacity to appoint a representative but the Tribunal believes that it is in the patient’s best interests for the patient to be represented’.^83^ Such a legal representative^84^ does not take their instructions from a litigation friend.^85^ The precise nature of their duties has been the subject of recent consideration by Charles J^86^ who agreed with an earlier Upper Tribunal judge^87^ that ‘a close analogy can be made between a legal representative appointed under Rule 11(7) for a patient who lacks capacity to give instructions on all relevant matters and that of a litigation friend appointed by the civil courts for a party’.^88^ In determining the obligations of a representative appointed by the Tribunal, Charles J therefore went on to consider cases such as *Re E*, *Masterman-Lister* and *RP*, concluding:

The appointment enables the solicitor to act for the patient in the proceedings and so seek his instructions and ascertain his views, wishes, feelings, beliefs and values. The best interests test in Rule 11(7)(b) and the general requirement to act in the best interests of a person who lacks relevant capacity mean that the legal representative is not only appointed in the patient's best interests but must also seek to promote them (having regard to the relevant issues of fact and law that are relevant in the proceedings).^89^

Charles J noted that he did not consider that the fact that the representative was appointed for the tribunal was thus acting for the tribunal (i.e. at the behest of and reporting solely to the tribunal) and thus in a different position to a legal representative appointed by a party. Accordingly, it was held that an appointed representative should not concede unarguable points if the party he represents either objected to or did not have capacity to consent to a concession; rather, such a representative should inform the tribunal that he is only advancing arguable points.^90^ He noted, however, that the relative informality of the tribunal ‘and its investigatory functions’ would enable it to hear directly from a patient as well as their representative, providing flexibility and ‘perhaps greater flexibility than in some or all courts’.^91^

### B. The Litigation Friend before the Court of Protection

The strands of practice and procedure discussed in the previous section have been drawn upon by judges of the Court of Protection to identify the duties upon litigation friends acting for P. Most materially, they have therefore:

1. Looked to litigation friends to fill the gap left by the absence of a children’s guardian under the FPR; and
2. And assumed that a litigation friend (as opposed to a lawyer instructed by that litigation friend, as to which see Section V below) is under a duty taken from the MCA 2005 not to advance a case on P’s behalf that is unarguable.

#### 1. ‘Filling the Guardian Gap’

The Court of Protection is regularly described as having (at least in part) an investigatory or inquisitorial, rather than adversarial, jurisdiction.^92^ To that end, it is empowered to call for a report from the Public Guardian or a Court of Protection Visitor^93^ or direct a local authority or NHS to provide a report upon such matters relating to P as the court may direct.^94^

However, there is no equivalent in the COPR to the role of children’s guardian found in the FPR. Especially in complex, ethically difficult or highly contentious cases, Court of Protection judges frequently—and understandably—require an independent assessment of the options that may be available.^95^ In the absence of a children’s guardian (one of whose functions is to advise the court upon the options available to it in respect of the child and the suitability of each such option including what order should be made in determining the application^96^), that assessment role would appear, by default, frequently to be pushed onto the litigation friend for P, especially where that litigation friend is the Official Solicitor.^97^ The current Official Solicitor, Alastair Pitblado, would appear to accept that role:

[I]n the Court of Protection the litigation concerns (1) whether P has or does not have capacity to make the relevant decisions; and (2) if not, what decisions are in P’s best interests. It is therefore necessary for me to put a case as to both to the court without … expecting to pre-empt the court’s determination on either.^98^ (emphasis added).

Court of Protection judges have emphasized the need for litigation friends to act dispassionately and independently^99^ in sifting and assessing the options available to P^100^ so as to be able to put a case to the court as to P’s capacity and—more often—the course of action that will be P’s best interests. It is extremely important to understand, however, that, while this role may very well be necessary, it is fundamentally incompatible with the role of ‘representation’ as conventionally understood. Indeed, the well-recognized tension between representation of the person (so-called ‘direct representation’) and representation of their interests (‘indirect representation’) underpins the division between the roles of children’s guardian and children’s directly instructed solicitor in those family proceedings where the child is considered able to instruct their own solicitor.

#### 2. The Best Interests Assumption

When acting on behalf of protected parties in civil or family litigation, as we have seen, litigation friends are not required to advance a case on behalf of a protected party that is not properly arguable. Such litigation friends owe duties not just to the party on whose behalf they act but also to the court.^101^ The duty to the court (as, it seems, an officer of the court^102^) would be not to advance that case—because such would be to frustrate the propriety and progress of the case.^103^

Both the current Official Solicitor and judges of the Court of Protection appear to consider that a litigation friend acting for P should proceed in a similar fashion. It is entirely understandable why this should be so, not least for the smooth functioning of the court system. However, it is important to recognize (as, in fairness, they do^104^) that this leads to a consequence where P’s litigation friend will not be advancing arguments that they know P strongly (if incapacitously) wishes to be advanced as to where their interests lie. They do so—it appears—on the basis of the following reasoning:

1. A litigation friend acts ‘for or on behalf of’ P^105^ in determining how to conduct the litigation, such that they are bound to follow the steps set down in section 4 MCA 2005.
2. Adopting a conception of ‘best interests’ contained in sections 1(5) and 4 MCA 2005 sufficiently wide to encompass assessing whether or not a particular outcome will be in P’s best interests and then deciding whether or not to advance a case on the basis of that assessment, *whether or not* that course of action is that which P wishes.

However, we suggest that this conclusion is, at best, questionable. If the litigation friend is acting under section 1(5) MCA 2005, there is a strongly arguable case that they would owe a duty to P (not to the court) to select the option that P would have chosen if such an option is available.^106^ Until and unless prevented by the deployment of appropriate case management tools by the court, a litigant with capacity can advance a hopeless case even if this would be most unwise. If it is right that a decision-maker must at least in some circumstances follow the wishes and feelings of P if it is practical to do so, a litigation friend acting on behalf of a person without capacity must advance such a hopeless case if that clearly reflects P’s wishes.

As matters stand, however, a litigation friend would not be encouraged to take this course of action by the judges of the Court of Protection, and indeed might be subjected to serious criticism for doing so.^107^ Further, and equally importantly given that the Official Solicitor is regarded as the litigation friend, the current Official Solicitor would undoubtedly not adopt such an approach.^108^ In so doing, he adopts a more fluid approach to the best interests test, where the wishes and feelings of P are less central to the analysis, and objective factors more central. Such a view is supported by jurisprudence in the Court of Protection and Court of Appeal,^109^ although it has been criticized in the academic literature^110^ and, as noted above, is arguably inconsistent with Supreme Court jurisprudence. Even on its own terms, this is not necessarily a licence to ignore the views of P. In *Re M, ITW v Z*, the court notes that the effect given to wishes and feelings will be determined by such things as the degree of P’s incapacity, the strength and consistency of P’s views, the impact on P of knowing that his or her views are not being given effect to, and the extent to which P’s wishes are ‘rational, sensible, responsible and pragmatically capable of sensible implementation’.^111^ These criteria are problematic in the present context, both on practical and theoretical grounds. Is it really within the spirit of the MCA for submissions to be made that we have every reason to believe P would not want, because he or she will not know that the prior wishes are not being given effect to? How is a litigation friend to decide when the criteria lead to different outcomes, as when wishes are clear, heart-felt and of long standing, but difficult to implement? Nonetheless, insofar as these factors are determinative, a litigation friend ought to give full effect to the wishes of P when the above criteria are met.

*M*, however, adds an additional factor: ‘crucially, the extent to which P’s wishes and feelings, if given effect to, can properly be accommodated within the court’s overall assessment of what is in his or her best interests.’^112^ This is a problematic comment. It cannot be a reference to the best interests test in the MCA, or it becomes circular. Equally, it cannot be a reference to objective best interests, since that would create a hierarchy of factors within the MCA best interests test, which is specifically what the case denies three paragraphs earlier. It is presumably a reference to that earlier statement that ‘the statute lays down no hierarchy as between the various factors which have to be borne in mind, beyond the overarching principle that what is determinative is the judicial evaluation of what is in P’s ‘best interests’’.^113^ As such it is a reminder that best interests is decided by the court, which under the existing Court of Protection jurisprudence is guided by but not bound by P’s wishes and feelings.

While that may be of assistance to the court, it is difficult to see that it assists litigation friends much: to what degree are such actors meant to second guess what the court’s view of best interests may be, and tailor their submissions accordingly? It is submitted that, to the extent that it is applied to the role of litigation friend, such speculation confuses the role of the litigation friend with that of the court. If Munby J’s point in *M* is that the court is not bound by P’s wishes and feelings, so be it; but that is a different question to whether actors representing P ought to advocate for P’s wishes and feelings, when the other *M* criteria are met.

There appear to be two other factors specific to the Official Solicitor which have led him to take the approach that he does (neither of which he has formulated publicly, so far as we are aware):

1. He is in a particular (unique) difficulty in those cases in which he is both litigation friend and lawyer for P.^114^ This peculiar combination would mean that, in some cases, he would be in the impossible position of—as litigation friend—instructing himself to run an argument that—as instructed lawyer—he would not able to do without breaching his professional obligations.^115^
2. As the Official Solicitor in modern times has always, himself, been a lawyer,^116^ it is more than likely than he—and his predecessors—have approached their task as litigation friend on the basis that they are discharging functions as a lawyer when so doing, and hence bound by the same obligations to the court as if they were a lawyer representing a client. It is not, in fact, obvious that this is (or necessarily should be) the case.^117^

Properly analysed, the first combination of factors does not hold true for other litigation friends. The second has not held true to date, although it will in due course do so for ALRs (see further Section V(B) below). However, because the Official Solicitor is regarded as the litigation friend *par excellence*, other ‘lay’ litigation friends understandably seek to model their approach on that he takes to cases, and the specific issues that affect his conduct have therefore had a much wider impact.

It is important to understand the consequences of this approach in real life. Perhaps the starkest example is the series of cases^118^ involving DD, a woman in her mid-thirties with diagnoses of autistic spectrum disorder and borderline learning disabilities. She had ‘an extraordinary and complex obstetric history’^119^ and was expecting her sixth baby. DD’s five children were all cared for by permanent substitute carers and four of the children had been adopted. DD had concealed some of her pregnancies, and it was clear that she did not wish antenatal assessment, medical interventions around birth, or post-natal examinations. The relevant medical bodies and the local authority sought declarations and orders in relation to the care and health of DD during the final stage of her current pregnancy, and in the safe delivery of the unborn baby, including declaration as to the lawfulness in arranging for DD’s baby to be delivered by planned caesarean section. As the proceedings unfolded, declarations were also sought as regards the conduct of an assessment of DD’s capacity to make decisions about contraception and also as to her sterilization. At all material stages, DD was represented by the Official Solicitor as her litigation friend who, on her behalf:

- consented to the initial application in relation to antenatal care and pre-birth scanning, ‘including—notably—the potential for deprivation of DD’s liberty and restraint in achieving the antenatal appointment’;^120^
- conceded that DD lacked the capacity to conduct the Court of Protection proceedings in circumstances where she had been a respondent to, and had to a limited extent participated in, four recent sets of (care order and placement order proceedings) without a litigation friend, and made a number of decisions relating to medical treatment in the past on the basis that she was capacitous;^121^
- ‘felt unable to make any recommendation on DD's behalf about the best interests of DD in relation to the mode of delivery of the unborn baby’;^122^
- initially opposed the proposed assessment of DD's capacity to make a decision about future contraception,^123^ but ultimately did not oppose steps to force entry for purposes of assessment of her capacity to make decisions in relation to capacity, although took steps to ensure that the evidence had been rigorously tested;^124^
- acknowledged on DD's behalf ‘not only that DD lacked capacity to make [a decision as to sterilisation] but that that sterilisation is indeed in DD's best interests’.^125^

DD’s case undoubtedly presented stark dilemmas for all concerned, and the Official Solicitor undoubtedly directed himself seriously and conscientiously to a consideration of DD’s interests before advancing the positions that he did. He also—very properly—sought to ensure that the evidence as to DD’s wishes and feelings were before the court. However, as in the earlier case of *E*,^126^ there was no-one present in the court room who actively *argued* DD’s case as it might properly be said DD would have wished it to be argued, and could properly expect it to be argued. In other words, there was no-one in the court room who actually *represented* DD, as opposed to (their conception of) DD’s interests.

The position is—if anything—potentially even more complicated in relation to challenges to deprivation of liberty. The ‘hard-edged’ nature of the obligations imposed by Article 5(4) ECHR was strongly emphasized by Baker J in *AJ (By Her Litigation Friend the Official Solicitor) v A Local Authority*,^127^ a case concerning the procedural requirements upon local authorities in authorizing deprivations of liberty in care homes (and hospitals) under Schedule A1 to the MCA 2005. Applying the same principles as those in play as regards detention under the Mental Health Act 1983,^128^ Baker J noted that, as ‘Article 5(4) gives AJ an unqualified right of access to the court […] there is no place in Article 5(4) for a best interests decision about the exercise of that right since that would potentially prevent the involvement of the court when, in Baroness Hale's words, ‘the whole point about human rights is their universal character’. On one view, this suggests a litigation friend must ensure that their client is put in a position *effectively* to challenge the circumstance of that deprivation of liberty, which means that there is arguably little or no room for ‘best interests’ determinations in the advancing of arguments testing the restrictions imposed upon P where it is clear that P wishes to challenge the deprivation of their liberty. Again, this puts the litigation friend in a very difficult—if not impossible—position where it is clear either: (1) that P has no hope in succeeding in their arguments; or (2) if P were to succeed, such would be fundamentally adverse to their well-being.

It therefore appears that—out of an understandable desire to focus proceedings on matters that are (objectively) arguable—the courts have imposed, and, almost more worryingly, the Official Solicitor has taken it upon himself to impose, a model of decision-making for litigation friends that takes it very far from the model of best interests decision-making that is applied to the *substantive* decisions that the court is being asked to take on behalf of P.

The final section of this article suggests how the duties upon litigation friends could be recast so as to seek to address, in a more principled fashion, the concerns that have led to the position set out in the preceding paragraphs. At this stage, we note that it would not solve all the problems that have been identified above if litigation friends were held not to take their duties from the MCA 2005—duties that, to reiterate, they owe to P and not to anyone else. It would, however, at least have the benefit of not leading to a misuse of the term ‘best interests’, and would be considerably more honest.

### C. The Court of Protection—Litigation Foes?

Drawing together the threads in the preceding section, we therefore suggest that the current position before the Court of Protection is deeply unsatisfactory in that:

- The court looks to P’s litigation friend not to represent P in any conventional sense, but to play a role which incorporates determination and relaying to the court of P’s wishes and feelings, investigation and detached assessment of options available to the court, and the presentation of what the litigation friend considers to be in P’s best interests. This role is, on a proper analysis, functionally identical to that of a children’s guardian in proceedings under the Children Act 1989.
- There are, in consequence, a number of cases in which it is clear that P’s litigation friend (most often, but not exclusively, the Official Solicitor) has either not positively advanced or indeed conceded matters where it is clear that, where P represented in the conventional sense, their representative would have taken a very different case. The consequence has been that no-one before the court has argued P’s corner for them.
- In practice, therefore, P faces two judges—one determining what substantive decisions to make on their behalf, and one deciding whether or not even to advance any arguments on their behalf as to those decisions.
- The combination of these factors leads dangerously close—if not over the line into—breaches of the fundamental procedural requirements identified in Section II above.

Before turning to our proposed solutions, consideration must be briefly given to the specific position of lawyers because the considerations binding upon them may offer part of the answer (even if, in the context of those acting as ALRs, they may prove a further part of the problem).

## V. LEGAL REPRESENTATION

### A. Lawyers Instructed by Litigation Friends

Contrary to popular belief, lawyers cannot necessarily advocate for what their clients pay them to advocate. Even hired guns owe responsibilities to others. Aside from the duty to the client, lawyers owe a duty to their opponent, to the court, to themselves, and to the State: ‘[t]o maintain a perfect poise amidst these various and sometimes conflicting claims is no easy feat.’^129^ Sir Thomas Bingham MR captured the issues at stake in *Ridehalgh v Horsefield*:

Legal representatives will, of course, whether barristers or solicitors, advise clients of the perceived weakness of their case and of the risk of failure. But clients are free to reject advice and insist that cases be litigated. It is rarely if ever safe for a court to assume that a hopeless case is being litigated on the advice of the lawyers involved. They are there to present the case; it is … for the judge and not the lawyers to judge it. It is, however, one thing for a legal representative to present, on instructions, a case which he regards as bound to fail; it is quite another to lend his assistance to proceedings which are an abuse of the process of the court … It is not entirely easy to distinguish by definition between the hopeless case and the case which amounts to an abuse of the process, but in practice it is not hard to say which is which and if there is doubt the legal representative is entitled to the benefit of it.^130^

Professional Codes of Conduct prevent lawyers advancing an entirely unarguable case.^131^ Counsel, for example, must not draft anything containing ‘any contention which you do not consider to be properly arguable’.^132^ What therefore is a lawyer to do if, because of delirium or advanced dementia, for example, P is adamant in their wish to leave a care home to return to the matrimonial home that was sold over 40 years ago? That contention is not properly arguable and it would be an abuse of the court’s process (and an abuse of P’s financial interests if the case was privately paid) for the lawyer to advocate for it. The same is true if the same instructions originate from the litigation friend for P.

This does beg the question: who is the lawyer’s client, P or the litigation friend? Section 87 of the Solicitors Act 1974 provides a non-exhaustive definition of the ‘client’ largely by reference to any person who as a principal, or on behalf of another person, retains or employs a solicitor and anyone who is or may be liable to pay the costs. In *Re EG*^133^ it was established that a solicitor is an agent acting on behalf of the patient rather than their receiver. More recently it has been suggested, *per curiam*, that, while a solicitor’s retainer is in one sense a personal contract, it is doubtful whether it generally requires instructions to be given by the client personally.^134^ Although the lawyer is the agent of P, rather than of P’s litigation friend, she must nevertheless take her instructions from a person capable of doing so, namely the litigation friend if instructed.

### B. Accredited Legal Representatives

In due course, assuming that a suitable scheme is set up, it will be possible for a lawyer to be appointed to represent P in proceedings without a litigation friend as an ALR. To the extent that their role is intended to mirror those of representatives appointed in the Mental Health Tribunal, it is very likely that the courts will seek to import the duties analysed at Section IV(A)(4). We suggest that there is considerable room to doubt whether those duties are capable of direct translation into the Court of Protection, in particular where they lead to arguments not being advanced on P’s behalf on a so-called ‘best interests’ basis.

It is also of note that there is an extensive literature from the USA, Australia, and Canada in particular as to the impossible position that lawyers are placed in when required to act as litigation friends for child clients.^135^ To the extent that ALRs will be placed in a functionally similar position for Ps, we suggest that exactly the same problems of principle will arise; their position will in some ways be even more acute because of the professional obligations upon ALRs—as lawyers—not to advance unarguable cases.

## VI. PROPOSALS FOR REFORM

This concluding section sets out where matters may go from here in terms of securing—insofar as possible—that ‘Ps’ are not merely objects but actually actors in proceedings before the Court of Protection.^136^ One obvious solution would be to amend the COPR to provide that, in every case in which P is joined, P is represented by a litigation friend (or ALR) and the equivalent of a children’s guardian is appointed. That would eliminate, at a stroke, many of the tensions identified above. However, this is unlikely to be realistic in an environment where resources are so limited. More achievable are amendments to the COPR and/or the accompanying Practice Direction to:

- Place a greater focus on identifying whether, in fact, P has capacity to conduct the proceedings. In this regard, some considerable assistance might be found for practitioners from The Advocates Gateway,^137^ and more broadly from the steps being taken in the Family Courts to implement lessons in relation to vulnerable witnesses in the criminal courts, lessons that are to be applied in the Family Courts also to parties who are ‘considered to be entitled to assistance on the grounds of […] incapacity’.^138^ Those steps (to be taken either on application or on the initiative of the court) include using communication devices and intermediaries.^139^ It would be entirely possible, for instance, for an intermediary to be instructed to assist in providing instructions.^140^
- Make clear that the primary duty of a litigation friend acting on behalf of P should—where P’s wishes and feelings can reliably be identified—be to proceed on the basis that the case that they put to the court is derived from those wishes and feelings.^141^ In other words, the task of the litigation friend acting for P is to *represent* P, not their conception of P’s interests or best interests.^142^ As a corollary, the litigation friend should be under an express duty (which is arguably already implicit from section 4(4) MCA 2005 if a litigation friend is to be considered as acting under section 1(5)) to support the person in expressing their wishes and feelings as regards the case that they wish to advance.
- Provide that, where the litigation friend considers that to advance the case that P can be identified as wishing to advance would be fundamentally adverse to P’s interests, then the litigation friend should invite the court to appoint an *amicus curiae* to discharge the function of assessing the options advanced both by P and the other parties to the proceedings.^143^ The litigation friend can then discharge their function of representing P.
- Provide that, if the litigation friend simply *cannot* identify reliable wishes and feelings upon which to advance a case to the court on P’s behalf, and it is not possible properly to derive assistance from their values and beliefs as to what course of action they would have wished to advance, then the litigation friend should seek to promote the course that least restricts their rights and freedoms.
- Make express any duty that the litigation friend may owe to the court in terms of the conduct of the proceedings. If there is really to be any such duty, we suggest, that must be viewed as secondary to that owed to P.

Two observations on the practicalities of these suggested steps are worthy of note. The first is to reiterate the point made at Section V(A) that, wherever a litigation friend instructs solicitors or counsel, there will always be a ‘filter’ built into the ability of those lawyers to advance cases that they properly consider to be unarguable to the court by way of their professional obligations. Concerns as to the extent to which a shift towards ‘direct’ representation of P may lead to undue burdens on the court may well be overstated.

The second is that the court already has extensive case management powers.^144^ It can (and should) deploy those powers to identify the issues to the proceedings, those issues which need a full investigation and hearing, and those which do not, and the procedure to be followed in the case. To the extent that those acting as litigation friends consider their duties to be coloured by obligations to the court (or to the other parties), robust judicial case management should alleviate those concerns. This would also provide the opportunity to ensure that a litigation friend is prevented from running arguments based upon a conception of P’s wishes and feelings that will do nothing other than cost P money. Of course, overly robust case management could then lead to the stifling of P’s ability to participate properly, but it would at least not derive from a position where their own representative was (perceived to be) pulling their punches.

## VII. CONCLUSION

What we now call litigation friends have a very long, tortuous and curious history. In the Court of Protection, practitioners, the judiciary and, above all, the subjects of those proceedings, are not well-served by the legacies of that history, in particular because the lessons from that history have been applied in a haphazard fashion within the context of a court with a very specific remit and function. Importantly, litigation friends owe duties to their client that are not synonymous with those duties owed by the Official Solicitor. The Official Solicitor is also (at least in some cases) a legal representative and as such owes separate duties to the client as a result. The approach of the Official Solicitor to his role as litigation friend (even if correct) should not therefore necessarily determine the ‘pure’ functions of the (lay) litigation friend.

It is time as the MCA reaches its decade to cast the legacy aside and to think more creatively so as to ensure that P is actually represented, not re-presented, by those purportedly acting on their behalf. This article has identified at least some of the tools which can be used in the collective endeavour that lies ahead; tools that may transform the litigation foe into litigation friend.

## Footnotes

1 In material part, Rule 6 of the Court of Protection Rules 2007 (‘COPR’) provides that ‘‘P’ means any person (other than a protected party) who lacks or, so far as consistent with the context, is alleged to lack capacity to make a decision or decisions in relation to any matter that is the subject of an application to the court and references to a person who lacks capacity are to be construed in accordance with the Act’. Note that the COPR are to be entirely rewritten during the course of 2016 to 2017 with all rules renumbered.

2 More controversially, it would appear that P does not need to be joined to most ‘uncontroversial’ applications for orders authorizing deprivation of liberty outside care homes and hospitals: *Re NRA & Ors* [2015] EWCOP 59, *Re JM and others* [2016] EWCOP 15.

3 Discussed in, *inter alia*, L Series, ‘The participation of the relevant person in proceedings in the Court of Protection: A briefing paper on international human rights requirements’, available at < http://sites.cardiff.ac.uk/wccop> accessed 26 November 2015; a further, more detailed discussion paper, drawing on work funded by the Nuffield Foundation, is anticipated as at the date of writing.

4 COPR r.3A(4)(3)(a).

5 *Re NRA & Ors* [2015] EWCOP 59, para 127–135.

6 In due course, it is anticipated that suitably experienced lawyers can be accredited and appointed—by the court—to act directly for P under the provisions of COPR r.3A(4)(b). They are modelled upon representatives appointed by the Mental Health Tribunal (see further Section IV.4).

7 COPR r.3A(4)(a).

8 COPR r.3A(4)(b); COPR r.3A(2)(b) provides that an ALR can be appointed ‘to represent P in the proceedings and to discharge such other functions as the court may direct’. It is not clear what other tasks an ALR who is representing P as a party can also discharge.

9 *Re M: ITW v Z and M* [2011] 1 WLR 344, COP para 35.

10 Eg, *London Borough of Hillingdon v Neary* [2011] 4 All ER 584, COP, *CC and KK v STCC* [2012] COPLR 627, COP, para 25.

11 See R Masterman and S Wheatle, ‘A Common Law Resurgence in Rights Protection?’ (2015) EHRLR 57.

12 [2014] AC 1115 at paras 67–71. See also *Kennedy v Information Commissioner* [2014] 2 WLR 808; *Re BBC* [2014] 2 WLR 1243.

13 *Osborn* at para 68.

14 *Shtukaturov v Russia* (2012) 54 EHRR 27 at para 64; *DD v Lithuania* [2012] MHLR 209 at para 114.

15 *Ivinovic v Croatia* [2014] ECHR 964 at para 35.

16 These decisions were made in the context of regimes which provide for ‘incapacitation’. The MCA 2005—functionally—provides for (at a minimum) partial incapacitation because a declaration that an individual lacks decision-making capacity in one or more domains is a necessary precursor to the Court of Protection taking the decision(s) on their behalf.

17 *B v Romania (No 2)* [2013] ECHR 393 at para 86.

18 *Shtukaturov v Russia* (2012) 54 EHRR 27 at para 68; *RP v United Kingdom* at para 65; *Seal v United Kingdom* (2012) 54 EHRR 6 at para 75.

19 See *MS v Croatia* [2013] ECHR 378 and *Ivinovic v Croatia* [2014] ECHR 964 and the discussion in Series: 2015. It would appear that the function of a guardian ad litem in the proceedings in issue in both *MS* and *Ivnovic* was to represent the individual’s rights and interests: see *MS* at para 44.

20 *Ivinovic* at para 45. See also *AJ (By Her Litigation Friend the Official Solicitor) v A Local Authority* [2015] COPLR 167, COP at para 35. In *YA*, Charles J expressed the view that *Ivinovic* did not ‘found a conclusion (which would be contrary to that reached in other cases) that States must ensure that in every case mentally disabled persons are afforded independent representation’: *YA v Central and North West London NHS Trust & Ors* [2015] UKUT 0037 (AAC) at para 41.

21 *MH v United Kingdom* (2014) 58 EHRR 35 at para 93 (emphasis added). See also *Winterwerp v The Netherlands* (1979) 2 EHRR 387 at para 60.

22 *RP v United Kingdom* [2013] 1 FLR 744. See in this regard the discussion of this case in L Series, ‘Legal Capacity and Participation in Litigation: Recent Developments in the European Court of Human Rights’ in G Quinn, L Waddington, and E Flynn (eds), *European Yearbook of Disability Law* (Martinus Nijhoff 2015).

23 CRPD Committee: General Comment 1, Art 12: Equal Recognition before the Law (April 2014).

24 CRPD Committee, Guidelines on Art 14 (September 2015).

25 See, eg, Freeman and others, ‘Reversing Hard Won Victories in the Name of Human Rights: A Critique of the General Comment on Article 12 of the UN Convention on the Rights of Persons with Disabilities’ (2015) 2(9) Lancet 844–50; *Torture in Healthcare Settings: Reflections on the Special Rapporteur on Torture’s 2013 Thematic Report* (Centre for Human Rights and Humanitarian Law, American University Washington College of Law 2014), available at <http://antitorture.org/wp-content/uploads/2014/03/PDF_Torture_in_Healthcare_Publication.pdf> accessed 7 December 2015.

26 COPR r.141(1).

27 COPR r.147 allowed an application to be brought by P, his litigation friend, or another party to the proceedings for the discharge of the appointment of the litigation friend where P ceased to lack the capacity to conduct the proceedings. Para 20 of Practice Direction 17A to the COPR provided that any such application had to be supported by evidence that P now had that capacity.

28 The old rule 141(1) has been abolished. COPR r. 3A(4) now provides that, unless P has capacity to conduct the proceedings, an order joining P as a party will only take effect where either P has a litigation friend or on or after the appointment of an ALR.

29 See, in particular, *Sheffield City Council v E & Anor* [2005] Fam 326, Fam Div at para 49.

30 See, eg, *Re SB; A Patient; Capacity to Consent to Termination* [2013] COPLR 445, COP, in which SB directly instructed lawyers in relation to the question of whether she had capacity to consent to a termination.

31 As the Law Society put it at para 4.1 of the 2015 iteration of their Practice Note on Representation before Mental Health Tribunals <http://www.lawsociety.org.uk/support-services/advice/practice-notes/mental-health-tribunals/> accessed 26 November 2015: ‘[t]he information that a patient is required to understand to instruct a solicitor in the context of an application to a tribunal is not complex and people severely affected by a mental disorder may still be able to provide instructions if you explain matters simply and clearly.’ Charles J in *AMA v Greater Manchester West Mental Health NHS Foundation Trust and Others* [2015] UKUT 36 (AAC) expressed the view that capacity to give instructions in relation to more complex matters (in that case, whether to withdraw an application) may require a higher threshold: see paras 38–42.

32 After its ‘discovery’ in 1989 by the House of Lords in *Re F (Mental Patient: Sterilisation)* [1990] 2 AC 1.

33 Which was, in fact, not a court at all, but rather the title of an office of the Supreme Court of Judicature (as it then was).

34 Department of Constitutional Affairs, ‘Draft Court Rules: Mental Capacity Act 2005 Court of Protection Rules’, Consultation Paper CP 10/06 (17 July 2006) (‘DCA Consultation Paper’), para 4.7. It is perhaps striking that the Law Commission’s consultation paper and report in the mid-1990s which laid the groundwork for the MCA 2005 contained no discussion at all of the party status of the person concerned in the sections on the role of the courts.

35 DCA Consultation Paper, para 4.7.

36 Under s 49 MCA 2005.

37 DCA Consultation Paper, para 4.7.

38 COPR r.140(1)(a) in respect of litigation friends; COPR r.147 (ALRs). There is—deliberately—no express requirement that an ALR have no interest adverse to that of P; mirroring, it would appear, the absence of any such requirement in relation to representatives appointed in Mental Health Tribunals, the ALR scheme taking its impetus from that regime.

39 The Court of Appeal has had two opportunities to consider the role of litigation friends; on both occasions it has declined to do so on the basis of unrelated technicalities: *TA v AA & Ors* [2013] EWCA Civ 1661 and *Re X (Court of Protection Practice)* [2015] COPLR 582, COP.

40 See, most recently, the decision in *Re NRA* [2015] EWCOP 59, in which Charles J drew without discrimination between authorities from civil, family and Mental Health Tribunal proceedings in order to consider the question of who could appropriately be appointed as litigation friend.

41 Prior to the enactment of the Civil Procedure Rules 1998 (‘CPR’), a distinction was drawn between a ‘next friend’, who acted on behalf on a plaintiff (now claimant), and a ‘guardian ad litem’ (so called, originally, to differentiate their role from that of a guardian of the person or of their estate) who acted on behalf of a defendant (or, in some contexts, a respondent). The relevant court rules now all refer to ‘litigation friend’, without distinction between the status of the party on whose behalf they act. A party to civil proceedings who requires a litigation friend because they lack the capacity to conduct those proceedings is now called a ‘protected party’ (CPR r.21(2)(d)) and the same also applies in relation to: (1) such adult parties in family proceedings (FPR r. 2.3 and Part 15); (2) such adult parties in proceedings before the Court of Protection other than the person concerned (COPR r.6). The functional equivalent of a litigation friend is provided for in insolvency proceedings in Part 7 of the Insolvency Rules 1986; the basis upon such individuals are appointed is somewhat different.

42 In England; the Mental Health Review Tribunal in Wales.

43 Dating back (at least) as far as the mid-seventeenth century: see *Offley v Jenney and Baker* (1647) 3 Chan Rep 92; 21 ER 738 and the discussion in J Seymour, ‘Parens Patriae and Wardship Powers: Their Nature and Origins’ (1995) 14 Oxford J Legal Stud 159–88, at 174.

44 Which were, essentially, identical as regards children or those with a mental disability as regards the discharge of the functions of next friends/guardians *ad litem*. A useful summary of the position as it pertained at the start of the twentieth century can be found in SE Williams and F Guthrie-Smith, *Daniell’s Chancery Practice* (8th edn, Stevens & Son 1914), which has regularly been cited in subsequent cases as providing assistance in understanding the roles and functions of litigation friends (see, for instance, *Re E (mental health patient)* [1984] 1 WLR 320, COP).

45 See eg *In Re Taylor’s Application* [1972] QB 369 at 381H per Lord Denning MR, in which he endorsed the proposition established in *Rhodes v Swithinbank* that (there) a next friend was an officer of the court.

46 [1984] 1 WLR 320, COP. The judgment was reversed on appeal ([1985] 1 WLR 245, CA), but these dicta were not questioned.

47 This reflected a long-standing principle of common law: *Murray v Sitwell* [1902] WN 119 and *In Re Berry* [1903] WN 125. After a considerable period of uncertainty, in *Re NRA* [2015] EWCOP 59, Charles J held (in the context of proceedings before the Court of Protection, but construing the CPR) that a litigation friend does not need to act by a solicitor at paras 127–57.

48 Indeed, the editors of Daniell’s Chancery Practice noted in 1914 that (in the context of children) ‘as it seems that as a next friend is required for the benefit of the defendant in order that there might be a person answerable to him for costs, the defendant may waive this benefit; in which case the action may proceed without a next friend’ (page 360, citing *Ex p Brocklebank* (1877) 6 Ch D 358, CA).

49 [2003] 1 WLR 1511, CA.

50 Para 30.

51 Para 65.

52 Including, in particular, by the Supreme Court in *Dunhill v Burgin (Nos 1 and 2)* [2014] 1 WLR 933.

53 *White Book 2015* para 21.2.1 (electronic edn, Sweet & Maxwel).

54 Which provides that a person may act as a litigation friend if he can ‘fairly and competently conduct proceedings on behalf of the child or protected party’.

55 [2008] 2 FLR 1516, CA at para 5 of the Advice reproduced at Annex B to the judgment. The judgment was given in care proceedings; however, because the protected party was the mother, rather than the subject child, this case is considered under this head because—functionally—her position was identical to that of a protected party in civil litigation.

56 *RP v United Kingdom* [2013] 1 FLR 744.

57 See eg, the discussion by Ormrod J (giving the judgment of the Court of Appeal) in *In Re L (An Infant)* [1968] P. 119, CA at 134 (the role of a *guardian ad litem* in relation to a child whose paternity was in dispute in divorce proceedings, giving rise to the exercise of the parental jurisdiction of the High Court).

58 Adoption of Children Act 1926.

59 Children Act 1975.

60 A useful summary of the development of the statutory provisions in this regard can be found in SAM Cooper, ‘Representing the Child: the Evolution of the Guardian ad litem in Care Proceedings’ (1993), Durham theses, Durham University (available at Durham E-Theses Online: <http://etheses.dur.ac.uk/5682/> accessed 26 November 2015).

61 Part 16.

62 Who will be an officer of Children and Family Family Court Advisory Service (‘Cafcass’) in England and Cafcass Cymru in Wales. The core functions of these two bodies are to be found in s 12 Criminal Justice and Courts Act 2000 and s 35 Children Act 2004 respectively.

63 Unless the court is satisfied that is it not necessary to do so in order to safeguard the child’s interests: s 41(1) Children Act 1989.

64 Where proceedings are specified, rules for the appointment, role and duties of the children’s guardian are contained within Ch 6 of Part 12 of the FPR. Where the proceedings are non-specified proceedings the appointment, role, and duties of the children’s guardian are covered within Ch 7.

65 Practice Direction 16A to the FPR, para 6.1 (in relation to specified proceedings); these apply in non-specified proceedings where the children’s guardian is a Cafcass/Cafcass CYMRU officer. A court considering any question with respect to a child under the Children Act 1989 may also request a report upon matters relating to that’s child welfare (s 7 Children Act 1989); it can also do so in relation to proceedings under the inherent jurisdiction.

66 Practice Direction 16A to the FPR, para 6.6.

67 See Hershman and McFarlane, *Children Law and Practice* (Jordans 2015) para 2708, and ss 41(1) and (2) Children Act 1989.

68 FPR r.16.29(2) provides that a solicitor is bound by the instructions of a child if the solicitor considers, having taken account of the views of the children’s guardian and any direction of the court under FPR r 16.21, that the child wishes to give instructions which conflict with those of the children’s guardian and that the child is able, having regard to his understanding, to give instructions on his own behalf.

69 FPR Practice Direction 16A, para 2.1.

70 See, in particular, J Fortin, *Children’s Rights and the Developing Law* (3rd edn, CUP 2009).

71 *R (R & Ors (Minors)) v CAFCASS* [2012] 1 WLR 811 at para 38.

72 At least public law proceedings such as care proceedings, ie those with the strongest analogy to welfare proceedings before the Court of Protection.

73 The Official Solicitor is now appointed by the Lord Chancellor under s 90 Senior Courts Act 1981.

74 Statement of Alastair Pitblado, Official Solicitor to the Senior Courts, reproduced as a supplement to the judgment of the Court of Appeal in *RP v Nottingham City Council and the Official Solicitor (Mental Capacity of Parent)* [2008] 2 FLR 1516, CA.

75 The precise basis upon which he is willing to act has evolved over time and is not relevant for present purposes. Regular Practice Notes have been issued by the Official Solicitor indicating the basis upon which he will act in various proceedings: see, most recently, that dated March 2013 relating to family proceedings and proceedings under the inherent jurisdiction in relation to adults <https://www.gov.uk/government/uploads/system/uploads/attachment_data/file/355822/ospt-practice-note.pdf> accessed 27 November 2015.

76 Or a child other than the subject of the proceedings.

77 [2008] 2 FLR 1516 (care proceedings in which the mother of the child lacked litigation capacity and was a ‘protected party’), see para 129 in particular. ‘The first and overriding duty of a litigation friend is to conduct the proceedings ‘fairly and competently.’ […] A litigation friend is a great deal more than the protected person’s advocate. On the premise that RP required a litigation friend, a number of difficult decisions needed to be taken – and taken objectively.’

78 [1982] 1 WLR 438, CA at 442.

79 *Airedale NHS Trust v Bland* [1993] AC 789 at 861 per Lord Goff of Chieveley.

80 Indeed, he on occasion play more than one role at once: in *In Re F (Mental Patient: Sterilisation*) [1990] 2 3AC 1, he appeared both in his role as Official Solicitor appearing *ex officio* as a party in the proceedings and, by separate Counsel, as advocate to the court.

81 And, separately, there are undoubtedly grounds to consider that he has had (not least through those who has chosen to instruct, most notably James Munby QC) had very considerable hidden influence upon the development of medical law: see J Montgomery, C Jones and H Biggs, ‘Hidden Law-Making in the Province of Medical Jurisprudence’ (2014) 77(3) MLR 343–78, and especially the discussion at 366–69. SC Manon, ‘Rights of water abstraction in the Common Law’ (1965) 83 LQR 47, 49–51.

82 In England (strictly, the First Tier Tribunal (Health, Education and Social Care). The Mental Health Review Tribunal has an equivalent power in Wales under rule 13 of the Mental Health Review Tribunal Wales Rules (SI 2008/2705).

83 Rule 11(7)(b) of the Tribunal Procedure (First-tier Tribunal) (Health, Education and Social Care Chamber) Rules (SI 2008/2699).

84 Who must be a member of the Law Society’s Mental Health Panel.

85 And the Official Solicitor does not—as a matter of policy—act as litigation friend for the patient in Tribunal proceedings.

86 *YA v Central and North West London NHS Trust & Ors* [2015] UKUT 37 (AAC); see also *AMA v Greater Manchester West Mental Health NHS Foundation Trust and Others* [2015] UKUT 36 (AAC).

87 Upper Tribunal Judge Rowland in *AA v Cheshire and Wirral Partnership NHS Foundation Trust* [2009] MHLR 308.

88 *YA* at para 82. In the next paragraph, Charles J acknowledged that there may be some differences between the roles, but considered that they should be addressed as and when they arose. As at the point of the hearing of the case, it had been anticipated that the Court of Appeal would be examining the role of litigation friends in the Court of Protection; it did not do so.

89 *YA* at para 93. It is unclear whether Charles J by ‘best interests’ was intending to refer here to the term as defined in the MCA (the term in Rule 11(7) does not cross-refer to the MCA).

90 *YA* at para 95.

91 *YA* at para 97. The same point was made in a different fashion in *AA v Cheshire and Wirral Partnership NHS Foundation Trust* [2009] MHLR 308, UT at para 23.

92 *Re G (Adult); London Borough of Redbridge v G, C and F* [2014] COPLR 416, COP see para 22. See also *Re NRA* [2015] EWCOP 59 at para 36, and the DCA Consultation Report (footnote 3) at paras 4.6–4.7.

93 See s 49(2) MCA 2005. Panels of such Visitors are established under s 61 MCA 2005; they can either be General or Special, the latter having medical (usually psychiatric) qualifications.

94 See ss 49(3) and (4) MCA 2005.

95 See, for instance, *Re N* [2015] EWCOP 76, a complex serious medical treatment case, in which Hayden J expressed his concern that, following a change of position by the Official Solicitor on behalf of P to support the application, ‘[t]he Court was then left in the position of contemplating a serious and important development in the evolution of the case law in the absence of opposing arguments. I was instinctively uncomfortable with that situation,’ and required the Official Solicitor ‘to act, in effect, as amicus to the Court, testing the evidence as it evolved’ (para 64).

96 Practice Direction 16A to the FPR, para 16.6.

97 We should emphasize that we do not here or in our critique of the current position more generally intend to cast doubt upon the *bona fides* of the Official Solicitor or the conscientiousness with which he approaches his role. Rather, in highlighting those cases in which decisions he has taken seem—to us—to jar, we intend to highlight the ad hoc and unsatisfactory nature of the system within which he operates.

98 See A Ruck Keene (ed), *Court of Protection Handbook* (Legal Action Group 2014) para 12.5, citing from a statement made by the Official Solicitor for purposes of judicial review proceedings. The Official Solicitor also makes clear in the same extract that this is a different role to that which he plays in acting for a protected party: ‘[i]n contradistinction, where I am acting as litigation friend for a protected party in the Court of Protection, my duty is to conduct the proceedings in the protected party’s best interests but not to seek to advance a case about the protected party’s best interests (as opposed to P’s best interests). I conduct separate and distinct exercises when acting as litigation friend for both P and a protected party (or child).’

99 *Re NRA* [2015] EWCOP 59 at para 170.

100 And, on occasion, investigating further what options may in fact also be available see *A and B (Court of Protection: Delay and Costs)* [2015] COPLR 1, COP at para 16: ‘The role of the litigation friend in representing P’s interests is not merely a passive one, discharged by critiquing other peoples’ efforts. *Where he considers it in his client's interest, he is entitled to research and present any realistic alternatives*’ (emphasis added).

101 They may, even, owe some form of duty to the other party in terms of the protection of the other party from the consequences of the individual’s lack of capacity. We are addressing here the position of litigation friends; the position of lawyers instructed by those litigation friends is addressed in the next section.

102 There have been no reported cases since the coming into force of the CPR upon the question of whether a litigation friend is still to be considered an officer of the court, although the authors of the current (9th) edition of *Halsbury’s Laws* consider that this to be the case (at least in relation to children in civil litigation). See para 1315, in relation to a litigation friend appointed to act on behalf of a child in civil litigation, citing (inter alia) *Rhodes v Swithinbank*.

103 Whether this approach is really justifiable is addressed in G Ashton, ‘Mental Challenges’ (2012) 162 NLJ 1495.

104 See *Re NRA* [2015] EWCOP 59 at para 170.

105 See s 1(5) MCA 2005.

106 See *Aintree University Hospitals NHS Foundation Trust v James* [2013] 3 WLR 1299, SC at para 45 per Lady Hale and A Ruck Keene and C Auckland, ‘More Presumptions Please: Wishes, Feelings and Best Interests Decision-Making’ (2015) Eld L J 5(3) 293–301. See also the provisional proposal by the Law Commission of England and Wales to amend s 4 MCA 2005 to introduce a rebuttable presumption that P’s wishes and feelings should determine what decision is in their best interests: *Mental Capacity and Deprivation of Liberty: A Consultation Paper* (Law Com Consultation Paper No 222, ch 12).

107 *Y Local Authority v PM and MZ* [2013] EWHC 4020 (COP) and most recently *Re NRA* [2015] EWCOP 59 at paras 158 and 170.

108 ‘A litigation friend cannot be acting in accordance with their duty, imposed both by the common law and the MCA 2005, to act in the relevant person’s best interests, if, on the one hand, they merely ‘rubber stamp’ the assessor’s assessment or, on the other, simply follow the person’s wishes and feelings’: A Pitblado, ‘An Unanswered Question: *TA v AA*’ (2014) Eld L J 4(1) 98–99.

109 See *RB v Brighton and Hove City Council* [2014] COPLR 629, CA; *K v LBX & Ors* [2016] COPLR 411, CA; *Re M; ITW v Z* [2011] 1 WLR 344, COP.

110 See, eg, R Harding, ‘The Rise of Statutory Wills and the Limits of Best Interests Decision-Making in Inheritance’ (2015) 78(6) MLR 945–70; P Bartlett and R Sandland, *Mental Health Law: Policy and Practice* (4th edn, OUP 2014) 188–93; R Barton-Hanson, ‘Sterilization of Men with Intellectual Disabilities: Whose Best Interest is it Anyway?’ (2015) 15(1) Med L Int 49–73; A Ruck Keene and C Auckland, ‘More Presumptions Please?’ above; D Wang. ‘Mental Capacity Act, Anorexia Nervosa and the Choice between Life-Prolonging Treatment and Palliative Care: A NHS Foundation Trust v Ms X’ (2015) 78(5) MLR 871–82; N Munro, ‘Taking Wishes and Feelings Seriously: The View of People Lacking Capacity in Court of Protection Decision-Making’ (2014) 36(1) J Social Welfare Family L 59–75.

111 [2011] 1 WLR 344, COP, at para 35.

112 [2009] EWHC 2525 (Fam), at para 35.

113 [2009] EWHC 2525 (Fam), at para 32.

114 In serious medical treatment cases, he almost invariably does not instruct external solicitors.

115 See further Section V.

116 Although he does not have to be: s 90 Senior Courts Act 1981, by which he holds his office, makes specific provision for the grant of the right to conduct litigation to holders of the position who are not otherwise authorized to do so: see s 90(3A)).

117 There is only reported case in which a lawyer—in that case the brother—acted as litigation friend for P: *AVS (By His Litigation Friend, CS) v NHS Foundation Trust and B PCT* [2011] COPLR Con Vol 219, CA. In that case, the brother brought the application in P’s name. The Court of Appeal did not judge his actions as litigation friend by reference to the fact he was a lawyer: see para 28.

118 [2014] EWCOP 8 (antenatal care and pre-birth scanning); the manner and location of delivery of the baby (caesarean section in hospital) ([2014] EWCOP 11); the administration of short-term contraception at delivery, and education about future contraception ([2014] EWCOP 13); the administration of short-term contraception post-delivery ([2014] EWCOP 44); and sterilization [2015] EWCOP 4.

119 *The Mental Health Trust & Ors v DD & BC* [2014] EWCOP 11 at para 1.

120 [2014] EWCOP 11 at para 40.

121 [2014] EWCOP 11 at para 66.

122 [2014] EWCOP 11 at para 14.

123 [2014] EWCOP 11 at para 14.

124 [2014] EWCOP 13 at paras 41 and 48.

125 [2015] EWCOP 4 at para 13.

126 *A Local Authority v E and Others* [2012] COPLR 441 [2012] COPLR 441, COP, concerning the case of a woman with severe anorexia who had sought to make an advance decision to tube feeding. See para 38: ‘[t]he Official Solicitor relies on the advice of Dr Glover. Dr Glover's preliminary impression after meeting E was that it was not in her interests to be forcibly fed. On reflection, and having studied her medical records, he advised that treatment which might return E to relatively normal life is available but has not so far been tried, and that she should receive it. The Official Solicitor accordingly sought a declaration that forcible feeding is in E's best interests.’

127 [2015] COPLR 167, COP.

128 In particular *Waite v UK* (2003) 36 EHRR 54.

129 Lord Macmillan, *Law and Other Things* (CUP 2015) 185. See also *Rondel v Worsley* [1966] 3 WLR 950, 962 *per* Lord Denning.

130 [1994] Ch 205, 234.

131 The relevant duty imposed upon solicitors is that imposed by the fourth (mandatory) principle under the SRA Code of Conduct 2011, and the duty upon barristers that imposed by para 303(a) of the Bar Code of Conduct.

132 Legal Services Act 2007 s 188; BSB Code of Conduct rC3, rC4, rC9.

133 [1914] 1 Ch 927, 83 LJ Ch 586.

134 *Blankley v Central Manchester Hospitals NHS Trust* [2015] EWCA Civ 4307, paras 37, 39, 43, 44.

135 See for particularly thought-provoking examples, G Monahan, ‘Autonomy *vs* Beneficence: Ethics and the Representation of Children and Young Persons in Legal Proceedings’ (2008) 8 Queensland U Tech L & Just J 3924–13; and S Malempati, ‘Ethics, Advocacy and the Child Client’ (2013–14) 12 Cardozo Pub L Pol’y & Ethics J 633––68. The Ilinois Supreme Court decided in *People v Austin M* 975 N.E.2d 22 (Ill. 2012) that a child in juvenile delinquency proceedings had been denied his Constitutionally mandated right to counsel where his attorney had been directed to act on a guardian ad litem (ie for these purposes, litigation friend) basis: see the discussion in A Bernabe, ‘A Good Step in the Right Direction: Illinois Eliminates the Conflict between Attorneys and Guardians’ (2013–14) 38 J Legal Prof 161–74.

136 In this section, we have derived considerable assistance from E Flynn, *Disabled Justice* (Ashgate 2015). We also have had regard to the discussion by the Australian Law Reform Commission of the role of ‘litigation representatives’ in ch 13 of their report upon Equality, Capacity and Disability in Commonwealth Laws (ALRC Report 124) <http://www.alrc.gov.au/publications/disability-dp81> accessed 26 November 2015. Finally, we have also noted that a system that relies upon the lawyer instructed for the equivalent of ‘P’ taking their role, first and foremost, to be an advocate for the person concerned and to advance the wishes of the person, whether or not they accord with what may seem to be his or her best interests, has been in place in New Zealand for many years under the Protection of Personal and Property Rights Act 1998 (see the analysis of the New Zealand High Court in *CMS v The Public Trust* [2008] NZHC 29 at paras 27 and 28).

137 <www.theadvocatesgateway.org>, which includes toolkits for advocates (including the identification of vulnerability in witnesses and defendants) and specialist guidance on particular conditions such as autism.

138 Proposed r 3B.1 to the FPR, being consulted upon at the time of writing.

139 Proposed r 3B(5)(a)(ij) and (iv).

140 Note that, if it is really the case that litigation friends act on P’s behalf for purposes of s 1(5), the corollary is, as discussed at Section III, s 1(3) MCA 2005 dictates that all practicable steps are taken to support the person to take the decisions—here as to the conduct of the proceedings—prior to moving to substituted decision-making.

141 As Lady Hale recognized in *Aintree University Hospitals NHS Foundation Trust v James* [2013] 3 WLR 1299, SC, at para 45, it is not always possible to determine what an incapable person’s wishes are. There are also difficult questions that may arise where the person concerned has never been in a position to express their views as to a relevant matter.

142 Whether this should also be the task of the litigation friend acting for others before the Court of Protection or protected parties in other litigation might be a different question.

143 The Court of Protection undoubtedly has the power to appoint an amicus: see, for instance, the role played by the Official Solicitor in the *Re X* proceedings concerning procedures for the lawful authorization by judges of deprivation of liberty. While the Court of Appeal was critical of the proceedings ([2015] COPLR 582, CA), there was no suggestion from their judgment that Sir James Munby P did not have the power to invite the Official Solicitor to act.

144 In particular in COPR r.5. Those case management powers are being further strengthened at the time of writing.

